# The efficacy of platelet-rich plasma in arthroscopic rotator cuff repair: A protocol of randomized controlled trial

**DOI:** 10.1097/MD.0000000000023232

**Published:** 2020-12-04

**Authors:** Yi Xue, Tong Lu, Yue Xu, Xi Cao

**Affiliations:** Department of Orthopedics, Changshu Hospital Affiliated to Nanjing University of Chinese Medicine, Jiangsu, China.

**Keywords:** arthroscopic rotator cuff repair, platelet-rich plasma, randomized, study protocol

## Abstract

**Background:**

Platelet-rich plasma (PRP), an autologous platelet concentrate (contain a large number of growth factors), has been widely investigated in healing and rebuilding the bone and tendon tissue. The objective of this prospective randomized research is to study and then compare the long-term effectiveness of the repair of arthroscopic rotator cuff without and with the platelet-rich plasma. It is assumed that there is no difference in the clinical results between patients receiving the repair of arthroscopic rotator cuff and the patients who do not receive PRP enhancement.

**Methods:**

This current study is a prospective, single-center, controlled, and randomized experiment. This study was reviewed and permitted via the institutional review committee of our hospital. All the patients will receive the written informed consent in order to involve in our clinical experiment. Patients were selected from the patients who received the repair of arthroscopic rotator cuff. Patients who meet the following conditions will be included in this study: ages ranges from 18 to 55; patients with complete tear of rotator cuff confirmed during operation; the patients agreed to wear the abduction stent for 4 weeks after operation; the preoperative count of platelet count is >150,000. All patients were evaluated at follow-up and baseline for the scores of Constant-Murley (CM) and American Shoulder and Elbow Surgeons (ASES), the numerical rating scale (NRS), and retear rate. The analysis is implemented with the SPSS 16.0 (SPSS Inc., Chicago, IL), the significance level remain at *P* < .05.

**Conclusions:**

The results of this study will provide useful new information on whether PRP is effective in the arthroscopic rotator cuff repair patients.

**Trial registration:**

This study protocol was registered in Research Registry (researchregistry6108).

## 1 Introduction

With the continuous development and advances of surgical instruments and technology, open technology is gradually being replaced by arthroscopic repair, which can recover faster and obtain good cosmetic results in rotator cuff repair.^[1–3]^ Nevertheless, about 27% of cases will recur.^[4]^ The insufficient tendon-bone healing is regarded to be a significant cause of recurrence. Rotator cuff retear may also be affected via a number of factors, containing smoking, the index of body mass, age, tear size, type, location, the quality of tendon, surgical technique employed, fatty infiltration, and postoperative rehabilitation.^[5,6]^ Many complementary biologic therapies have been utilized for the improvement of the early tendon healing quality, and they may also decrease the incidence of the late tendon sequelae.^[7]^

Platelet-rich plasma (PRP), an autologous platelet concentrate (contain a large number of growth factors), has been widely investigated in healing and rebuilding the bone and tendon tissue.^[8–11]^ These growth factors possess a significant effect on the regeneration, repair, and acceleration of biochemical processes, thereby reducing pain caused by the tears of rotator cuff and promoting the healing of the rotator cuff.^[12–16]^ Many systematic reviews and meta-analysis have shown that the use of the PRP in repairing the rotator cuff can not obviously improve clinical result, but it can improve the structural healing of small to moderate tears.^[17–20]^ Currently, many clinical trials, which assessed the influence of the PRP on repairing the rotator cuff, have generally reported short-term results.^[21–24]^ Therefore, the randomized controlled studies containing the long-term follow-up are critical for the evaluation of the whole latent benefits of PRP.

The objective of this prospective randomized research is to study and then compare the long-term effectiveness of the repair of arthroscopic rotator cuff without and with the platelet-rich plasma. It is assumed that there is no difference in the clinical results between patients receiving the repair of arthroscopic rotator cuff and the patients who do not receive PRP enhancement.

## 2 Material and method

### 2.1 Trial design

This current study is a prospective, single-center, controlled, and randomized experiment. The study protocol was approved by the ethics committee of Changshu Hospital Affiliated to Nanjing University of Chinese Medicine (K20200927). All the patients will receive the written informed consent in order to involve in our clinical experiment. The research protocol was registered in Research Registry, with the number researchregistry6108. Between October 2020 and October 2021, our agency will evaluate 100 eligible patients.

### 2.2 Participants

Patients were selected from the patients who received the repair of arthroscopic rotator cuff. Patients who meet the following conditions will be included in this study: ages ranges from 18 to 55; patients with complete tear of rotator cuff confirmed during operation; the patients agreed to wear the abduction stent for 4 weeks after operation; the preoperative count of platelet count is >150,000. Exclusion criteria include as following: patients who do not meet the inclusion criteria; previous shoulder surgery; patients with active infection, remote infection, sepsis, and osteomyelitis that may spread to surgical area; patients who are uncooperative or have a disease that prevents them from following instructions, or who do not want to come back for the follow-up examination.

### 2.3 Randomization

The randomization list was generated with the block randomized procedure. Participants were randomly allocated to one of two parallel combinations to receive either PRP or placebo (with the ratio of 1:1). In order to assign participants, an independent researcher who was not participated in surgery prepared a sealed and opaque envelope, which contains the treatment task. Subsequently, an uninformed researcher opened the envelope and assigned participants to either control group or PRP group on the basis of the chose index card.

### 2.4 PRP preparation

For the patients randomly divided into PRP group, the PRP was acquired with the GPS III Platelet Concentration System. In short, the PRP is prepared through taking blood (54 mL) from the patient, and then mixing the resulting blood with citrate anticoagulant (6 mL) into the disposable separation tube, afterwards, centrifuging it at 3200 rpm for 15 minutes. After the above centrifugation, the platelet-deficient plasma needs to be removed from centrifuge to generate the PRP (6 mL), the PRP is extracted and then utilized for the intraoperative injection.

### 2.5 Surgical management and PRP application

All the repairs of rotator cuff were implemented by a senior shoulder surgeon under the condition of general anesthesia with a lateral decubitus position. Participants receive surgery in the beach-chair position. In all cases, plastic surgery is performed and debridement is performed at the same time, and the larger nodules are not peeled off. The simple stitches and absorbable double-load suture fixator are utilized to carry out the single-row repair. In the case of partial tear, subluxation and complete dislocation, procedures are performed on the biceps tendon. After completing the arthroscopic surgery, patients in treatment group underwent the local activation of PRP. According to the adopted scheme, the ultimate volume of PRP (6 mL) can be acquired. Through applying lateral portal, spray applicator kit equipped with the autologous thrombin and PRP syringes is placed between repaired rotator cuff and the bone with no cannula. Subsequently, inflow is closed and then the fluid under the arthroscope is carefully inhaled through outflow cannula. The PRP are then slowly injected. In control group, the patients will be given the same dose of saline in the same method.

### 2.6 Postoperative protocol

The shoulder is fixed with the abduction bracket for 4 weeks. During fixation, the patients are encouraged to conduct the exercise of muscle contraction and to perform active and passive motions of the elbows, wrists, and fingers. After the patient is gradually weaned from the abduction brace from 4 to 6 weeks after surgery, further passive exercise (range of motion) and active auxiliary range of motion exercise are allowed. After 3 months, the patients begin to carry out the intensive exercises. After 3 months, jogging and other light sports are allowed, and 6 to 9 months later, the patients can fully resume exercise according to personal recovery.

### 2.7 Outcomes measures

All patients were evaluated at follow-up and baseline for the scores of Constant-Murley (CM) and American Shoulder and Elbow Surgeons (ASES), the numerical rating scale (NRS), and retear rate. The primary outcome is retear rate. The NRS of people are assessed for pain on an 11-point scale ranging from zero (for “no pain”) to 10 (for “most severe pain possible”). The ASES scores and CM scores are reliable, effective and responsive, and cover various activities of daily living to assess shoulder function, and the assessment are performed at baseline, 12 months, 24 months, and 48 months.

### 2.8 Sample size calculation

The power analysis was carried out previously for the determination of the size of the study sample to offer 80% statistical power at an alpha level of 0.05%. After calculation, in each group, the size of sample of 50 patients is enough to measure the difference of CM score by at least 5 points at the end of 4 years, and 10% of data may be lost. Therefore, a total of 110 participants (55 per group) are enlisted for this research.

### 2.9 Statistical analysis

The standard deviation and mean are the descriptive statistics. The normality of all the data was detected via the Kolmogorov–Smirnov test. The difference of value before and after the treatment was calculated by the paired sample *t* test. The student *t* test was applied for the comparison of variances between study group and control group. The analysis is implemented with the SPSS 16.0 (SPSS Inc., Chicago, IL), the significance level remain at *P* < .05.

## 3 Discussion

The objective of this prospective randomized research is to study and then compare the long-term effectiveness of the repair of arthroscopic rotator cuff without and with the platelet-rich plasma. It is assumed that there is no difference in the clinical results between patients receiving the repair of arthroscopic rotator cuff and the patients who do not receive PRP enhancement. One limitation of this experiment is that the quantity of growth factors and the number of platelets exist in the activated platelet-rich plasma was unknown. The results of this study will provide useful new information on whether PRP is effective in the arthroscopic rotator cuff repair patients.

## Author contributions

**Conceptualization:** Yi Xue, Tong Lu.

**Data curation:** Yi Xue, Tong Lu, Yue Xu.

**Formal analysis:** Yi Xue, Tong Lu, Yue Xu.

**Funding acquisition:** Yi Xue.

**Investigation:** Yi Xue, Tong Lu.

**Methodology:** Tong Lu, Xi Cao.

**Project administration:** Yi Xue.

**Resources:** Xi Cao.

**Software:** Tong Lu, Yue Xu, Xi Cao.

**Supervision:** Yi Xue, Xi Cao.

**Validation:** Yue Xu.

**Visualization:** Yue Xu.

**Writing – original draft:** Yi Xue, Tong Lu.

**Writing – review & editing:** Yue Xu, Xi Cao.
